# The Impact of Temperature, Humidity, and Sunshine on Internet Search Volumes Related to Psoriasis

**DOI:** 10.2196/49901

**Published:** 2023-10-19

**Authors:** Hakan Lane, Mark Walker

**Affiliations:** 1 Brandenburg Medical School Neuruppin Germany; 2 Department of the Natural and Built Environment Sheffield Hallam University Sheffield United Kingdom

**Keywords:** psoriasis, infodemiology, internet search, internet searching, web search, information seeking, information search behavior, information search behaviour, dermatology, skin, weather, temperature, humidity, sunshine

## Abstract

We examined internet searches on psoriasis in Germany and found that in weeks with high search volume, mean temperature and humidity were lower and sunshine level was higher compared to weeks with low search volume.

## Introduction

A connection between psoriasis severity and weather has long been suspected. Cold, dry weather has been noted anecdotally to exacerbate symptoms, whereas sunshine improves the condition [1]. Psoriasis is more prevalent in the northern hemisphere, possibly due to colder temperatures [2]. Studies examining internet search data show that searching is more common during winter than summer, suggesting a relationship with temperature [3]. A recent systematic review [4] presented inconclusive results; no seasonal changes were seen in half the studies examined and summertime improvement was found in only 30% of studies. Few studies have assessed specific weather features like temperature, humidity, and sunshine levels.

## Methods

In this study, we examined internet searches related to psoriasis in Germany, looking at whether patterns in searching were weather related. The internet search volume for the search term “psoriasis” (disease) for Germany was obtained from Google Trends [5]. This website provides the relative search volume (RSV) of specific search terms, with values representing the number of searches for a term relative to the total number of searches done. The week of maximum searching is assigned an RSV of 100 against which searching done during other weeks is calibrated. Weekly data from January 2018 to 2023 was downloaded.

Weather data from the German Wetterdienst (national meteorological service) was used. Daily data for mean temperature, percentage relative humidity, and total hours of sunshine was obtained for 6 weather stations: Berlin, Cologne, Frankfurt, Hamburg, Munich, and Stuttgart. Weekly mean values for temperature and humidity as well as total weekly hours of sunshine were calculated. A value for all of Germany was calculated using data from the 6 stations.

## Results

**Figure 1:**
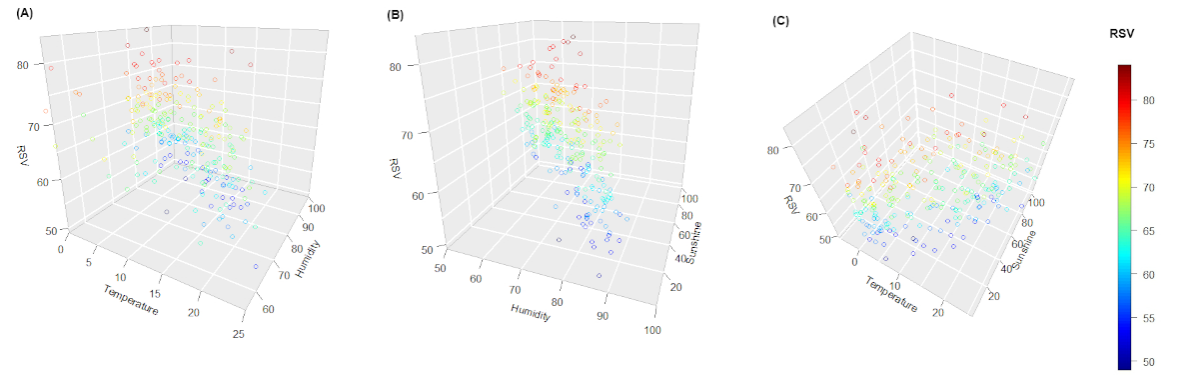
3D scatter plots showing the association between the weekly search volume of “psoriasis” and (A) temperature (°C) and humidity (%), (B) humidity and hours of sunshine, and (C) temperature and hours of sunshine. Data are for weekly mean values from select German weather stations. RSV: relative search volume.

The RSV ranged from 53 to 100. Although there was only a slight correlation between searching and specific weather features (temperature: ρ=–0.13, humidity: ρ=–0.23, sunshine: ρ=0.11), 3D scatter plots suggested differences between weeks of high and low searching. Low search weeks (blue in Figure 1A) occurred across greater temperature ranges than high search weeks (red in Figure 1A). Most high search weeks occurred when temperatures were low. Similarly, low search weeks occurred across a greater range of humidity levels than high search weeks, which occurred more frequently at low humidity levels (Figure 1B). Given comparable levels of sunshine, high search weeks occurred at lower temperatures than low search weeks (Figure 1C).

Notable differences were apparent when comparing mean values between the lowest (RSV≤60; n=43) and highest (RSV≥70; n=86) search weeks. The mean temperature was higher during low rather than high search weeks (low: 11.18±7.00 °C; high: 8.60±6.30 °C), as was humidity (low: 79.73±9.12%; high: 71.25±9.99%), but sunshine hours was lower (low: 27.61±21.64 hours; high: 36.48±24.46 hours). The difference for humidity was significant (2-tailed *t* test: *P*<.001).

These trends were further explored using linear regression. Since the search data were not normally distributed (Shapiro-Wilk W=0.96; *P*<.001), they were logged. Separate regressions were performed with logged values for temperature, humidity, and sunshine as dependent variables. The resulting coefficients indicated that higher temperature and humidity led to lower search volumes but more sunshine was associated with increased searching (temperature: –0.01, humidity: –0.13, *P*<.001; sunshine: 0.01, *P*=.04). Regression fitting suggests an RSV of 78 at 10 °C, declining to an RSV of 40 at 15 °C. At 65% humidity, an RSV of 93 is expected; when humidity rises to 80%, the RSV should drop to 47. With 40 hours of sunshine per week, an RSV of 68 is expected.

## Discussion

This study revealed that weeks of high internet searching related to psoriasis were associated with cold, dry weather conditions. Differences were most apparent when comparing the highest and lowest search weeks. The possible association between high searching and more sunshine is of interest; this is contrary to anecdotal evidence but supports recent survey work [4], which underlines that the factors influencing psoriasis are multifactorial. More extensive research to ascertain the influence of specific weather factors is required.

Google Trends data allowed for the examination of search volume trends over long time periods and on a weekly basis. However, only national data were available, meaning regional assessment was not possible. Local differences may exist. We assumed that internet searching is constant throughout the year, but increases in winter months may occur when people spend more time on computers due to longer, darker evenings. Future studies could control for this problem by calibrating search volume with results from nonseasonal conditions. Another assumption is that internet searching reflects the severity of psoriasis. One would expect this to be the case because people are more likely to search when their symptoms worsen and discomfort is felt. It is well known that internet searching reflects the incidence of a condition [6]. The effects of weather are unlikely to be apparent in clinical settings.

## Abbreviations

RSV: relative search volume
